# Molecular identification and mycotoxins analysis of some fungal isolates from postharvest decayed apple in Qena, Egypt

**DOI:** 10.1186/s12870-025-07205-2

**Published:** 2025-09-11

**Authors:** Abdelrahman Saleem, Amany Atta El-Shahir

**Affiliations:** Department of Botany and Microbiology, Faculty of Science, South Valley University, Qena, 83523 Egypt

**Keywords:** Apple, *Penicillium*, ITS genes, Mycotoxins, HPLC analysis

## Abstract

**Background:**

Apples are important for human nutrition because these provide vital nutrients, including vitamins and minerals, that are needed for a balanced diet. A suitable environment for the growth and survival of various microorganisms is also provided by multiple nutrients, such as carbohydrates, minerals, vitamins, and amino acids. *Penicillium* spp. are the cause of blue mold, one of the most common postharvest apple fruit diseases. Using morphological description and ITS gene sequencing, the current study aimed to identify *Penicillium* isolates associated with infected apple. High-performance liquid chromatography (HPLC) was used to demonstrate these isolates' capability for producing mycotoxins.

**Results:**

The initial identification of fungi was based on micromorphological characteristics, growth texture, and colony patterns. *Penicillium* (35.01%) and *Talaromyces* (15.62%) were the most prevalent fungal genera. These isolates' morphological identity was validated by ITS gene amplification and sequencing. The generated fragments' ITS gene sequencing values were 477, 480, 510, 478, and 478 bps for *Penicillium expansum* strain AP1, *Penicillium crustosum* strain AP2, *Talaromyces atroroseus* strain AP3, *Penicillium expansum* strain AP4, and *Penicillium expansum* strain AP5, respectively. It was examined whether the five isolates could produce citrinin and patulin. The highest value of toxins was recorded for patulin (24180 ppb), that produced by *P. expansum* AP5*,* followed by *P. crustosum* AP2 (19360 ppb). However, the highest value for citrinin was 24890 ppb detected for *P. expansum* AP4, followed by *P. expansum* AP5 (19320 ppb).

**Conclusion:**

Blue mold caused by *Penicillium expansum*, *Penicillium crustosum*, and *Talaromyces atroroseus*. These isolates have the ability to produce citrinin and patulin with different degrees. So blue mold is one of the most harmful diseases in post-harvest apple fruits.

**Supplementary Information:**

The online version contains supplementary material available at 10.1186/s12870-025-07205-2.

## Background

Apple (*Malus domestica*) is a valuable fruit crop worldwide Wenneker et al. [73]. In the Mediterranean and Middle East regions. Fruits stored in cold storage have been found to exhibit a variety of damage, most notably rot signs Habib et al. [32]. Fruits are often kept for a long time. Fungal pathogen-induced diseases of postharvest fruits are a barrier to long-term preservation. Spoilage of post-harvest fruits is one of the most important topics. Fungal infection in stored fruits is easy to appear in warm and humid environments Gariepy et al. [31]. Fungal contamination of fruits, particularly post-harvest, poses a significant threat to food safety and market quality worldwide. Among various fruits, apples, oranges, and bananas are commonly affected, with apples frequently exhibiting higher levels of fungal colonization. Several species within the genus *Penicillium* are known to be major contributors to fruit spoilage, especially in apples, where species like *P. digitatum*, *P. expansum*, and *P. italicum* are often encountered [24, 59, 66]. Previous studies across different regions—including Saudi Arabia, Malaysia, and Lebanon [9], Hasan and Zanuddin 2020, [32] have highlighted the diversity of fungal genera associated with fruit spoilage, such as *Alternaria*, *Aspergillus*, *Cladosporium*, *Fusarium*, *Penicillium*, and *Rhizopus*. These investigations highlight the widespread occurrence of *Penicillium* as a dominant genus, often linked to the production of harmful mycotoxins like patulin. Notably, *P. expansum* has been repeatedly identified as the primary agent of blue mold in apples during cold storage, with substantial implications for both fruit quality and consumer health. Understanding the distribution and toxigenic potential of these fungi is essential for developing effective strategies to manage post-harvest losses and ensure food safety.

Mycotoxins are secondary metabolites released from filamentous fungi that naturally occur in food. The existence of such compounds in food series is of high significance for human health due to their severe toxicity effects at low dose levels. Mycotoxin pollution of fruits causes health hazards and economic losses. Many mycotoxins were found in fruits and their products, such as aflatoxins, ochratoxin, citrinin, patulin, and *Alternaria* toxins [25, 27, 28, 40, 43, 57, 58]. They have a fundamental concern for the food industry and consumers. Several genera of fungi, such as *Alternaria*, *Aspergillus*, *Botrytis*, *Cladosporium*, *Colletotrichum*, *Fusarium*, *Geotrichum*, *Penicillium,* and *Rhizopus* able to produce mycotoxins and cause diseases. Various fruits have variable shelf lives for spoilage. A strong correlation was observed between fungi and the country of origin of fruits. The two most prevalent mycotoxins that *Penicillium* species generate are citrinin (CTN) and patulin (PAT). They are frequently connected to fruits and their byproducts. Regular quality assessment necessitates the use of basic and reliable approaches for tracking the toxins in meals [21, 22]. The tiny lactone mycotoxins (patulin, penicillic acid, and moniliformin) were evaluated by Frisvad [30]. These mycotoxins were produced by numerous fungi. Twenty-nine species were assured for patulin secretion. The toxin was secreted by 3 *Aspergillus* spp., 3 *Paecilomyces* spp., 22 *Penicillium* spp., which were related to 7 *Penicillium* sections and one *Xylaria* sp. A polyketide lactone mycotoxin is called patulin (PAT). By harming essential organs like the liver, kidney, gastrointestinal tract, and brain, it has caused gastrointestinal, immunological, and neurological disorders in humans in addition to having a carcinogenic effect [14, 54, 55]. Following its initial detection from *Penicillium patulum* [16], a variety of *Aspergillus* and *Penicillium* species, including *A. clavatus*, *A. giganteus*, *A. terreus*, *P. claviforme*, *P. expansum*, *P. melinii*, and *P. patulum*, have been documented to secrete patulin. *P. expansum* is the primary exporter of patulin in fruits, such as apples and their derivatives, and is one of the species responsible for the deterioration of pomaceous fruits, which exhibits rapid soft rot and eventually blue pustules [22, 27, 41, 63]. Patulin has been recorded as a contaminant of numerous moldy infected fruits, with major contaminated sources of apples and apple products, and sometimes in other fruits such as apricots, peaches, grapes, and pears. The rotted parts of fruits are the major contaminated tissues [17, 19, 48, 53, 54]. In this respect, Ionescu et al. [41] reported that patulin (PAT) is a mycotoxin commonly produced by several fungal species, including *Aspergillus clavatus*, *Penicillium claviforme*, *P. expansum*, *P. patulum*, and *P. urticae*. It has been associated with various toxicological effects in animals, such as gastrointestinal disturbances (including hemorrhage, distension, and ulceration), as well as neurotoxic, immunotoxic, and genotoxic outcomes.

Recently, Hussain et al. [37] results confirmed that PAT contamination is of particular concern in fruit crops and their derived products, especially apples and grapes, where it may be present in significant concentrations. The detection and quantification of PAT in these commodities are frequently carried out using analytical methods like high-performance liquid chromatography (HPLC), underlining the importance of monitoring efforts in safeguarding public health.

PAT was widespread in fruits and derived products, including apples and apple-derived products in many countries of the world [4, 18, 40, 41, 46, 48, 53, 54, 63, 70, 72]. The hepatotoxic and nephrotoxic mycotoxin known as citrinin (CTN) was initially identified in *Penicillium citrinum* and other species of *Aspergillus*, *Monascus*, and *Penicillium* Xu et al. [74]. Long-stored foods such as cereals and cereal-based goods, as well as fruits, herbs, spices, and moldy cheeses, can include CTN as a contaminant [51]. The literature contains very little information about CTN toxicity. Research has demonstrated that in experimental animal models, exposure to CTN causes chromosomal abnormalities, hepatotoxicity, and nephrotoxicity [15, 34, 42, 62]. CTN has been identified as a possible risk factor for human Balkan endemic nephropathy, much like ochratoxin A [52]. However, due to insufficient data in animals, the International Agency for Research on Cancer [39] categorized this mycotoxin in category 3 as not classifiable as carcinogenic to humans.

The main objective of this research was to document the most common isolates related to apple-infected fruits. Isolates are identified using morphological descriptions and ITS gene sequencing. Using high-performance liquid chromatography analysis, these species'capability to produce PAT and CTN toxins was also examined.

## Materials and methods

### Collection of apple fruit samples

From January to August 2023, thirty samples of rotten yellow apple fruits were gathered at random from different markets in Qena, Egypt. The fruit samples were gathered in sterilized plastic bags, taken directly to the Mycology laboratory, Faculty of Science, South Valley University, Egypt, and kept at 4 °C till fungal analysis.

### Isolation of apple fruit fungi

The fungus was isolated using a culture medium termed potato-dextrose agar (PDA), which consists of 200 potatoes, 20 dextrose, and 15 g of agar (Merck, Darmstadt, Germany). The bacteriostatic agent used was chloramphenicol (0.05 g/L). On PDA medium, fungi associated to apple fruits were isolated. After 30 seconds of immersion in a 1% v/v sodium hypochlorite solution, the fruits were rinsed three times with sterile distilled water to get rid of any remaining sodium hypochlorite residue and dried with sterile filter paper. Fruit segments with the infection were chopped into 1 cm^2^ pieces. Each plate contained four pieces that were transferred onto the culture medium's surface. For every sample, three plates were made. For five to seven days, the plates were incubated at 25°C. The obtained fungi were counted, identified, and calculated as means per 12 segments. According to Domsch et al. [23], fungi were identified using morphological and microscopic descriptions.

### DNA extraction

Following the instructions that came with the QIAamp DNeasy Plant Mini kit, fungal cultures were cultivated on the PDA for seven days at 25 °C before being used for DNA extraction. For further processing, 100 milligrams of fungal mycelia were frozen at −80°C for 24 hours Sambrook et al. [60].

### Polymerase chain reaction and amplification of 5.8S rDNA

The amplification of 5.8S rDNA was performed using the universal primer pair ITS1

(50TCCGTAGGTGAACCTGCGG03) and ITS4 (50TCCTCCGCTTATTGATATGC03). The PCR included 35 cycles, with an initial denaturation of 5 min at 94 °C, secondary denaturation for 30 s at 94 °C, primer annealing for 40 s at 56 °C, primer extension for 45 s at 72 °C, and a final extension for 45 s at 72 °C. The reaction was made in a quantity of 25 µL containing 6 µL DNA templates, 12.5 µL master mix, and 1 µL (20 p mol) of each primer. The PCR products were electrophoresed (for 1 h at 80 V) in 1.0% agarose gel in Tris-borate-EDTA buffer at pH 8. The gel was stained with ethidium bromide and was observed in a gel documentation system. The PCR products were sequenced by Elim Biopharmaceuticals Inc. (a biotechnology company in Hayward, California, CA, USA). PCR product purification and sequencing (bidirectional sequencing) was performed as follows: QIAquick PCR product extraction kit (Qiagen Inc., Valencia, CA, USA) was used for the purification of the PCR product directly. A BigDye Terminator V3.1 cycle sequencing kit (Perkin-Elmer, Foster City, CA, USA) was used for performing sequencing using an Applied Biosystems 3130 genetic analyzer (Hitachi, Ltd., Tokyo, Japan). Centrisep (spin column): cat number CS-901 was used for 100 reactions. A purified PCR product was sequenced in the forward and reverse directions on an Applied Biosystems 3130 automated DNA Sequencer ABI, 3130 (Thermo Fisher Scientific Inc.,Waltham MA, USA). Using a ready-reaction BigDye Terminator V3.1 cycle sequencing kit (Perkin-Elmer/Applied Biosystems, Foster City, CA, USA). To demonstrate sequence identity to GenBank accessions, a BLAST® (http://blast.ncbi.nlm.nih.gov/Blast.cgi) analysis (Basic Local Alignment Search Tool) Altschule et al. [8] was first conducted after DNA sequences were acquired using an Applied Biosystems 3130 genetic analyzer (HITACHI, Japan). The Mega Align module of Lasergene DNAStar version 12.1 Thompson et al. [67] generated the phylogenetic tree, and phylogenetic analyses were conducted using MEGA6's maximum likelihood and maximum parsimony Tamura et al. [64].

### Determination of mycotoxin production

#### Cultivation and extraction procedures

To examine the production of mycotoxins, five isolates from infected apple fruit samples were grown in three duplicates in a 500 ml flask with 100 ml of potato-dextrose broth [22]. The flasks were shaken at 150 rpm in the dark for seven days while being incubated at 25°C. The cultures were then filtered through Whatman No. 1 filter paper 200 ml of filtrate and 100 ml of extraction solvent (chloroform: methanol, 2:1 v/v) were added. According to [72] the mixture was shaken for at least a minute. At 40 °C, rotary evaporation was used to separate the solvent. The residue was collected in 2 ml of methanol when it was almost dry and stored at 4 °C until HPLC analysis.

#### High-performance liquid chromatographic (HPLC) analysis of mycotoxins

HPLC-(Agilent 1100), which consists of two LC-pumps and a UV/Vis detector, was used to do the analysis. 150 mm × 4.6 mm C18 column with a particle size of 5 µm. The Agilent ChemStation was used to obtain and analyze chromatograms. Water, acetonitrile, and acetic acid (99:99:2 v/v, isocratic mode) make up the mobile phase. With the mobile phase, the detector was set at 276, and 331 nm for patulin and citrinin, respectively. The flow rate was adjusted to 1.0 mL/min. According to Davis and Dinier (1978), Tessini et al. [65], and Catana et al. [18], 10 µl of each sample was injected three times. The peaks that showed lamda max (ƛ_max_) values within the expected range for mycotoxins were processed by estimating their peak height and area after their resulting chromatograms were tested for mycotoxins (patulin and citrinin). The concentration of mycotoxins is directly correlated with the values of peak area and height. The measured mycotoxin concentration was expressed using the concentration in the culture broth of the isolates under investigation. Taguchi et al. [63]. The mycotoxins standard was acquired from Sigma Aldrich in the United States.

## Results

### Morphological identification of fungal isolates

On the PDA medium at 25 °C, six fungal species from four genera were isolated from diseased apple fruits. *Talaromyces* and *Penicillium* were the most prevalent fungal genera. *Penicillium* contributed 278 colonies of the total count of fungi, with a percentage of 35.01 and high occurrence remarks (26 out of 30 samples). While *Talaromyces atroroseus* contributed 124 colonies of the total count of fungi, with a percentage of 15.62 and high occurrence remarks (15 out of 30 samples) (Table 1).

**Table 1:** Total counts, (calculated per (3 replicates x4 pieces), number of cases of isolation (NCI, out of 30 samples), and occurrence remarks (OR) of fungal genera and species isolated from apple fruits

**Fungi**	**TC**	**%**	**NCI&OR**
*Aspergillus*	51	6.42	7 M
*A.flavus*	6	0.76	1 R
*A. niger*	45	5.67	6 L
*Penicillium*	278	35.01	26 H
*P. expansum* AP1	72	9.07	6 L
*P. crustosum* AP2	74	9.32	8 M
*P. expansum* AP4	42	5.29	4L
*P. expansum* AP5	90	11.34	8 M
*Rhizopus oryzae*	12	1.51	4 L
*Talaromyces atroroseus* AP3	124	15.62	15 H
Gross total count	794		
Number of genera	4		
Number of species	6		

*OR* Occurrence remarks (out of 30 samples), *H* high occurrence from 15-30 cases, *M* moderate occurrence from 7–14 cases, *L* low occurrence from 3–6 cases and R = rare occurrence from 1-2 cases [26].

The fungi were initially identified by their micromorphological characteristics, growth texture, and colony patterns. Following a 7-day incubation period at 25 °C on PDA media. The results revealed the presence of five isolates of *Talaromyces* and *Penicillium* (*P. expansum* AP1, *P. crustosum* AP2, *T. atroroseus* AP3, *P. expansum* AP4, *P. expansum* AP5). Colonies were blue-green for the first three isolates, white yellow, and white blue, respectively. The reverse of the colonies was yellow, yellow to cream, colorless, orange, and yellow, respectively. The average colony diameters were 4, 3, 2.6, 2, and 2.5 cm, respectively.

**Fig. 1:**
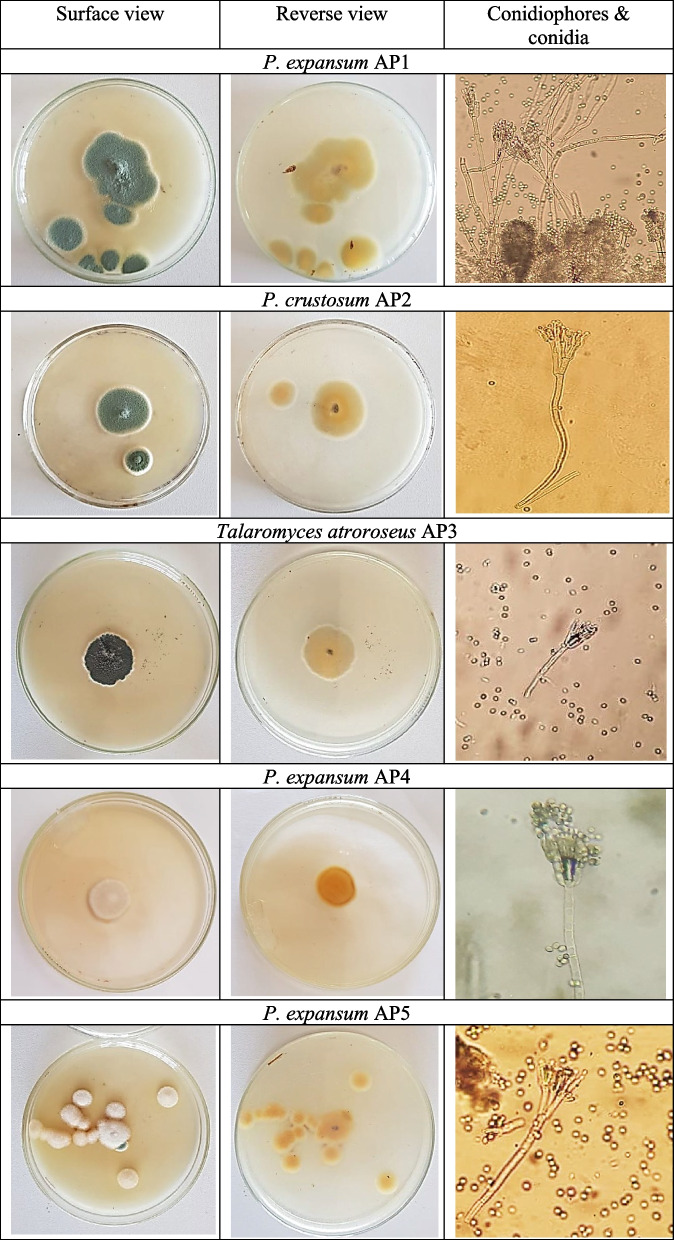
Morphological and microscopic features (surface view, reverse view, conidiophores and conidia) of different *Penicillium* and *Talaromyces* isolates isolated from infected apple fruits at 7-days old cultures on PDA medium at 25°C

The textures of the growth were fasciculate for the three *P. expansum* isolates. The texture of the other colonies was weakly fasciculate to crustose for *P. crustosum* AP2 and velutina to funiculose for *T. atroroseus* AP3. *P. expansum* AP1 conidiophores (19.6 X 3.9 µm) were smooth, asymmetrical, usually biverticillate, and had smooth walls. They had three to six metulae (12.5 X 2.5 µm) that bore phialides (11.5 X 2.5 µm). Conidia had a diameter of 3.3µm, were smooth, and were globose to subglobose. With clearly roughened walls, *P. crustosum* AP2 conidiophores (21.2 X 4.1 µm) were terverticillate and occasionally biverticillate. They had two to five metulae (14 X 3.8 µm) that bore phialides (10.1 X 3.1 µm) and smooth walls. Conidia had a diameter of 3.1µm, were smooth, and were globose to subglobose. Conidiophores of *T. atroroseus* AP3 (15.2 X 2.1 µm) were symmetrically biverticillate and smooth. They had three to eight metulae (9.2 X 2.6 µm) that bore phialides (8 X 2 µm) and smooth walls. Conidia had a diameter of 2.2 µm, were smooth, and were elliptical to subglobose*. P. expansum* AP4 conidiophores (17.6 X 2.9 µm) possessed two to five metulae (13.2 X 2.9 µm) and were rough and asymmetrically terverticillate. They produced 12.1 x 2.6 µm phialides. The globose, smooth conidia had a diameter of 3.8µm. Conidiophores of *P. expansum* AP5 (18.1 X 2.1 µm) were asymmetrically biverticillate and smooth. They had three to five metulae (15.1 X 3.3 µm) that bore phialides (13.8 X 3.1 µm) and smooth walls. Conidia were smooth, elliptical, and 4 µm in diameter (Fig. 1 and Table 2).

**Table 2:** Morphological description and measurement of different structures of *Penicillium* and *Talaromyces* species (averages of 10 *Penicilli* for each isolate) of 7-days old cultures on PDA medium at 25 °C

**Fungal isolates**	**Colony color**	**Colony reverse color**	**Colony diameter (cm)**	**Colony** **Texture**	**Conidiophore (µm)**	**Conidia (µm))**	**Metulae (µm))**	**Phialides (µm)**
*P. expansum* AP1	Blue green	yellow	4	Fasiculata	19.6 X 3.9	3.3	12.5 X 2.5	11.5 X 2.5
*P. crustosum* AP2	Blue green	Yellow to cream	3	Weakly fasciculate to crustose	21.2 X 4.1	3.1	14 X 3.8	10.1 X 3.1
*T. atroroseus* AP3	Blue green	colorless	2.6	Velutina to funiculose	15.2 X 2.1	2.2	9.2 X 2.6	8 X 2
*P. expansum* AP4	White yellow	orange	2	Fasiculata	17.6 X 2.9	3.8	13.2 X 2.9	12.1 X 2.6
*P. expansum* AP5	White blue	yellow	2.5	Fasiculata	18.1 X 2. 1	4	15.1 X 3.3	13.8 X 3.1

### Molecular Characterization of Penicillium Species Using ITS rDNA Gene Sequencing

**Fig. 2:**
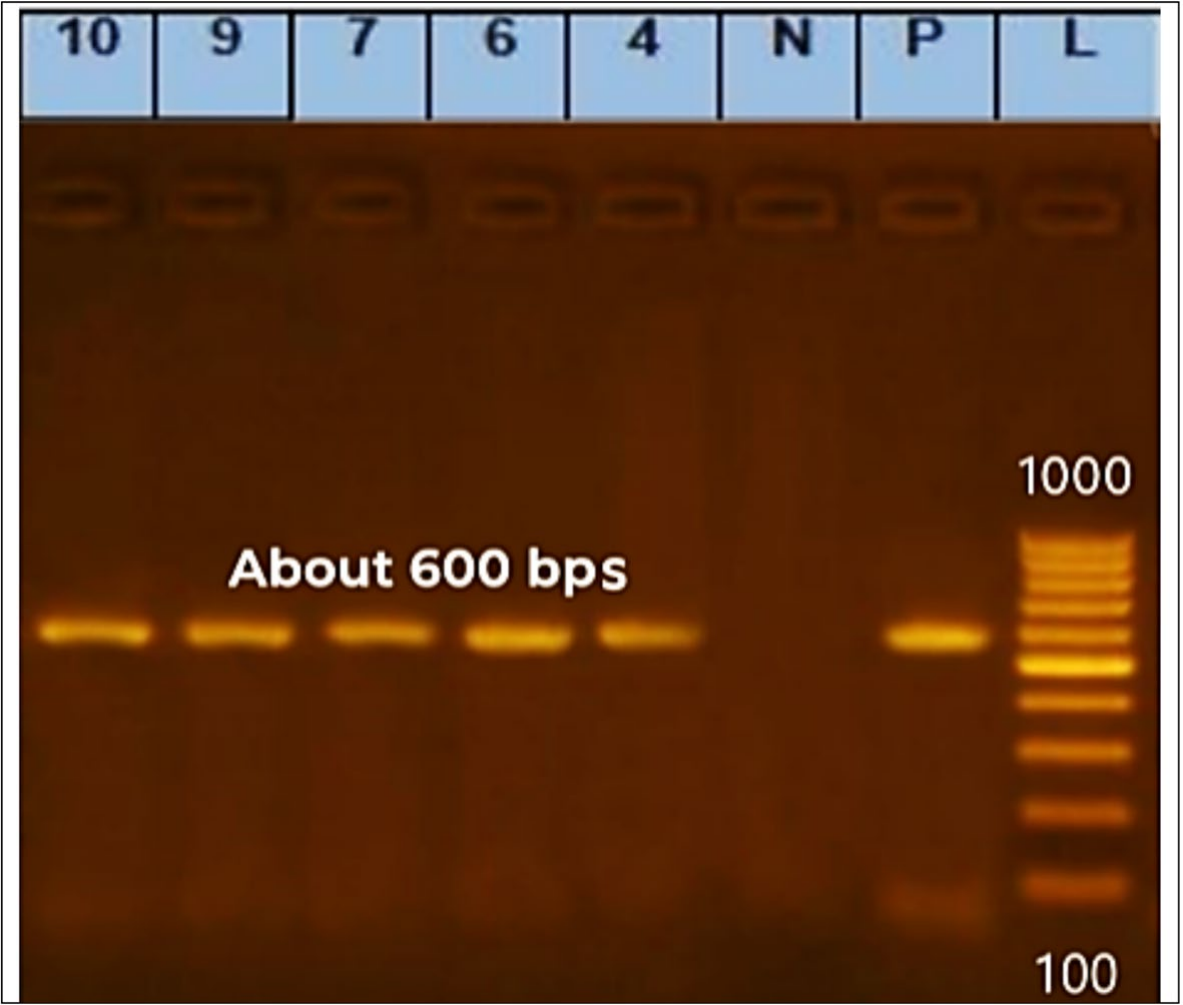
Agarose gel electrophoresis of PCR amplification of DNA products, PCR amplification of DNA products (~600 bps) using ITS1/ITS4 primer pair. L—ladder (100–1000 bps); P—positive control consists of a segment of DNA of known size (the same size as the target amplicon, shows that the primers have attached to the DNA strand); N—negative control: a sample without DNA, but contains all essential components of the amplification reaction show if contamination of the PCR experiment with foreign DNA has occurred; (4) *P. expansum* AP1, (6) *P. crustosum* AP2, (7) *T. atroroseus* AP3, (9) *P. expansum* AP4, (10) *P. expansum* AP5

The tested apple samples contained one isolate of *Talaromyces* and four isolates of *Penicillium*. To validate the morphological identification, these isolates were chosen for molecular identification by ITS1 and ITS4 rDNA gene sequencing. The designations assigned to the five isolates were *P. expansum* AP1, *P. crustosum* AP2, *T. atroroseus* AP3, *P. expansum* AP4, and *P. expansum* AP5. The ITS region was successfully amplified from the DNA of every isolate using the fungal-specific universal primer pairs ITS1 (forward) and ITS4 (reverse). Gel electrophoresis yielded fragment sizes around 600 bps (Fig. 2).

**Fig. 3:**
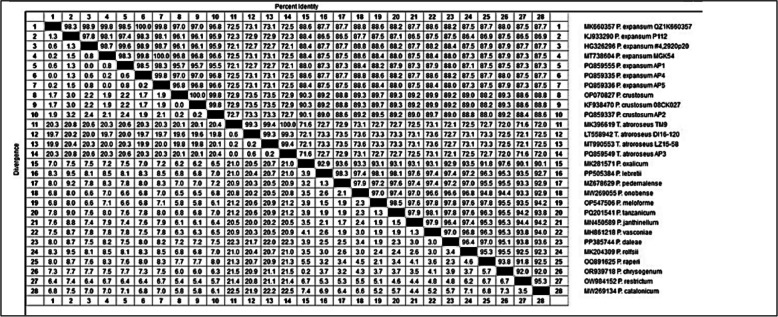
Sequence distance of *P. expansum* AP1, *P. crustosum* AP2, *T. atroroseus* AP3, *P. expansum* AP4, *P. expansum* AP5 showing identity range of 96.8–99.3% for different *Penicillium* and *Talaromyces* isolates

For *P. expansum* AP1, *P. crustosum* AP2, *T. atroroseus* AP3, *P. expansum* AP4, and *P. expansum* AP5, the obtained ITS gene sequencing results were 477, 480, 510, 478, and 478 bps. These fungi's morphological identity was validated by their molecular identification. The acquired rDNA sequences were analyzed by NCBI-BLAST. The ITS rDNA sequences were searched using BLAST to confirm the morphological identification. The NCBI GenBank database shows which isolates have the closest match (96.8–99.3% similarity) (Fig. 3).

**Fig. 4:**
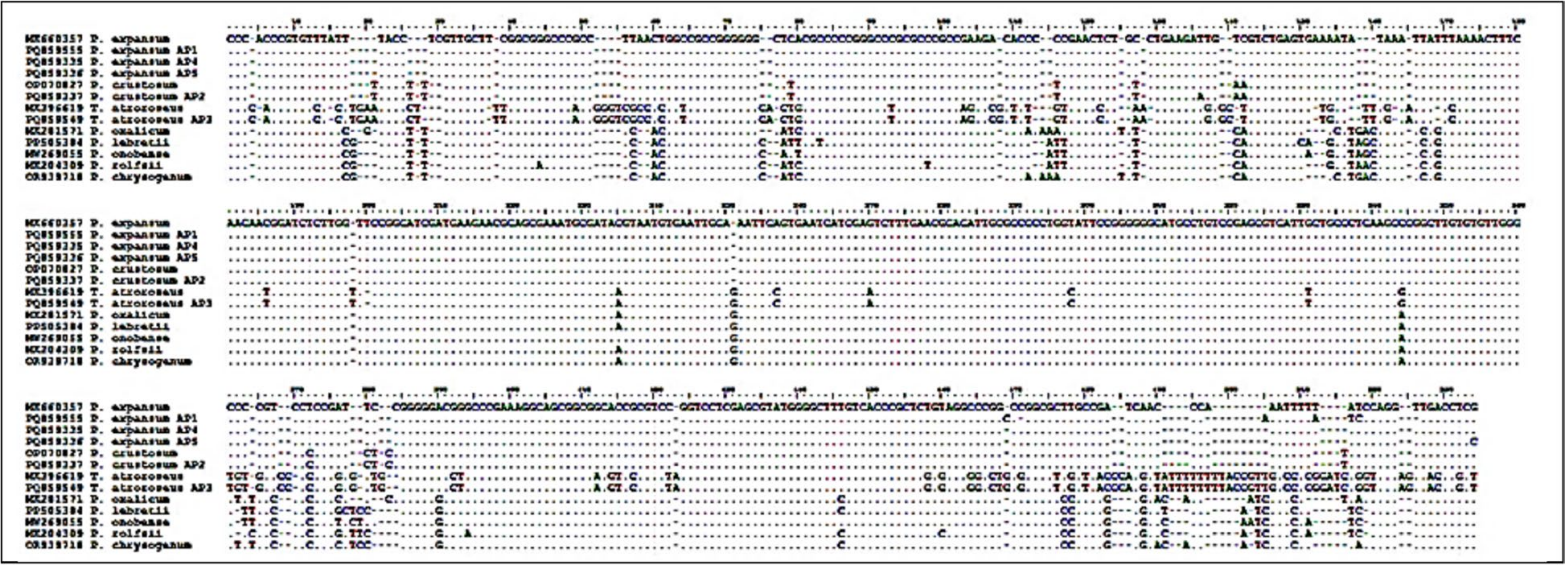
Nucleotide alignment report for all studied strains

All of the examined strains'nucleotide alignments revealed a high degree of homology between the strains under study and those that had the closest match similarities in the NCBI GenBank database (Fig. 4). ITS rDNA sequences from the five studied strains have been added to the NCBI GenBank databaseas follow *Penicillium expansum* strain AP1 (PQ859555), *Penicillium crustosum* strain AP2 (PQ859337), *Talaromyces atroroseus* strain AP3 (PQ859549), *Penicillium expansum* strain AP4 (PQ859335), and *Penicillium expansum* strain AP5 (PQ859336).

**Fig. 5:**
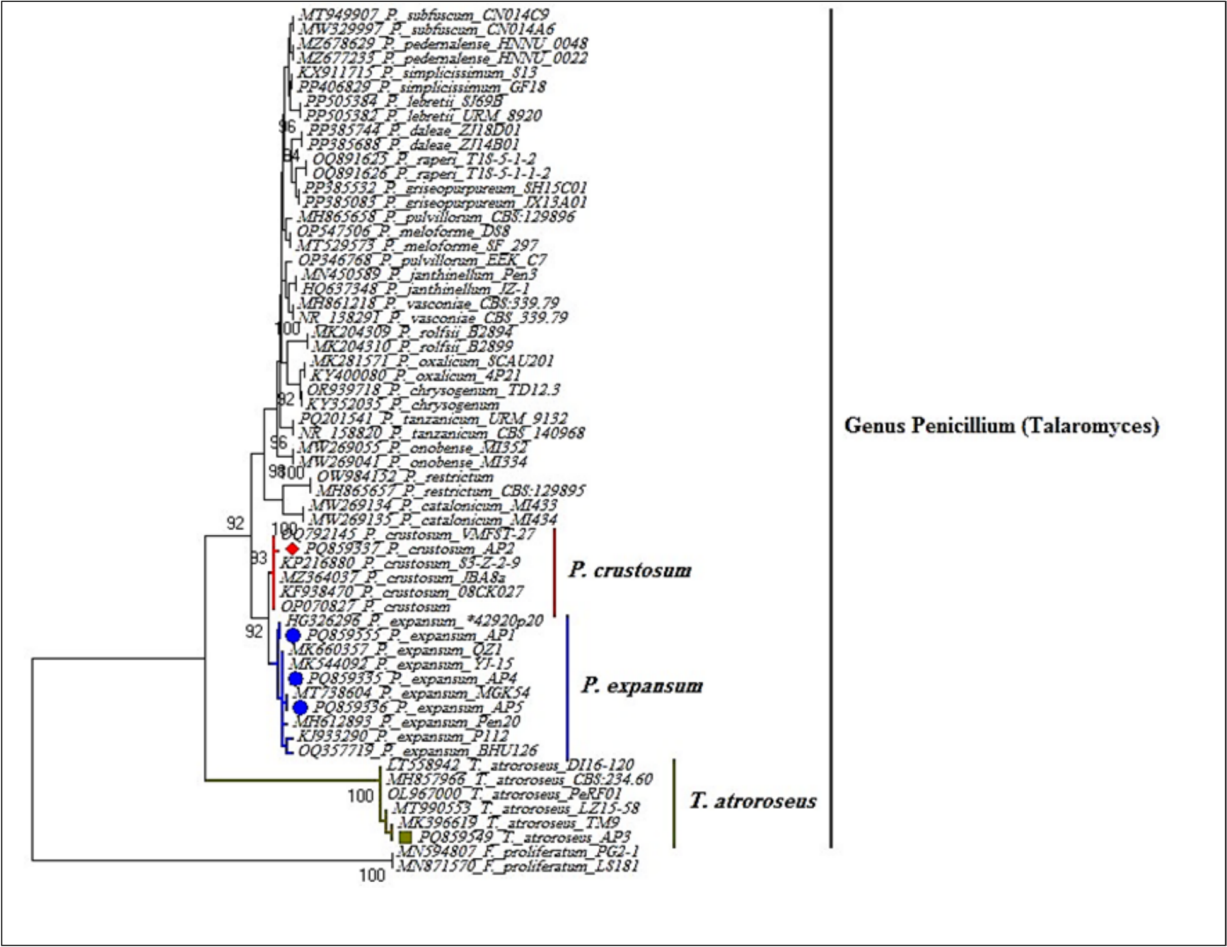
Phylogenetic tree generated from *Penicillium expansum* strain AP1 (PQ859555), *Penicillium crustosum* strain AP2 (PQ859337), *Talaromyces atroroseus* strain AP3 (PQ859549), *Penicillium expansum* strain AP4 (PQ859335), and *Penicillium expansum* strain AP5 (PQ859336). and its related species/isolates with sequences available in the GenBank database based on datasets of ITS rDNA gene sequences. Isolates with red, blue, and olive dots were recovered from apple fruits in this study

The investigated strains'variability was confirmed by the BLAST results. Three clusters are likely found in the phylogenetic tree of the five strains that were obtained. Cluster I contained *P. crustosum* strain AP2 (PQ859337), cluster II included three strains, *Penicillium expansum* strain AP1 (PQ859555), *Penicillium expansum* strain AP4 (PQ859335), and *Penicillium expansum* strain AP5 (PQ859336). However, the cluster III included *Talaromyces atroroseus* strain AP3 (PQ859549) (Fig. 5).

### Determination of mycotoxin production

**Fig. 6:**
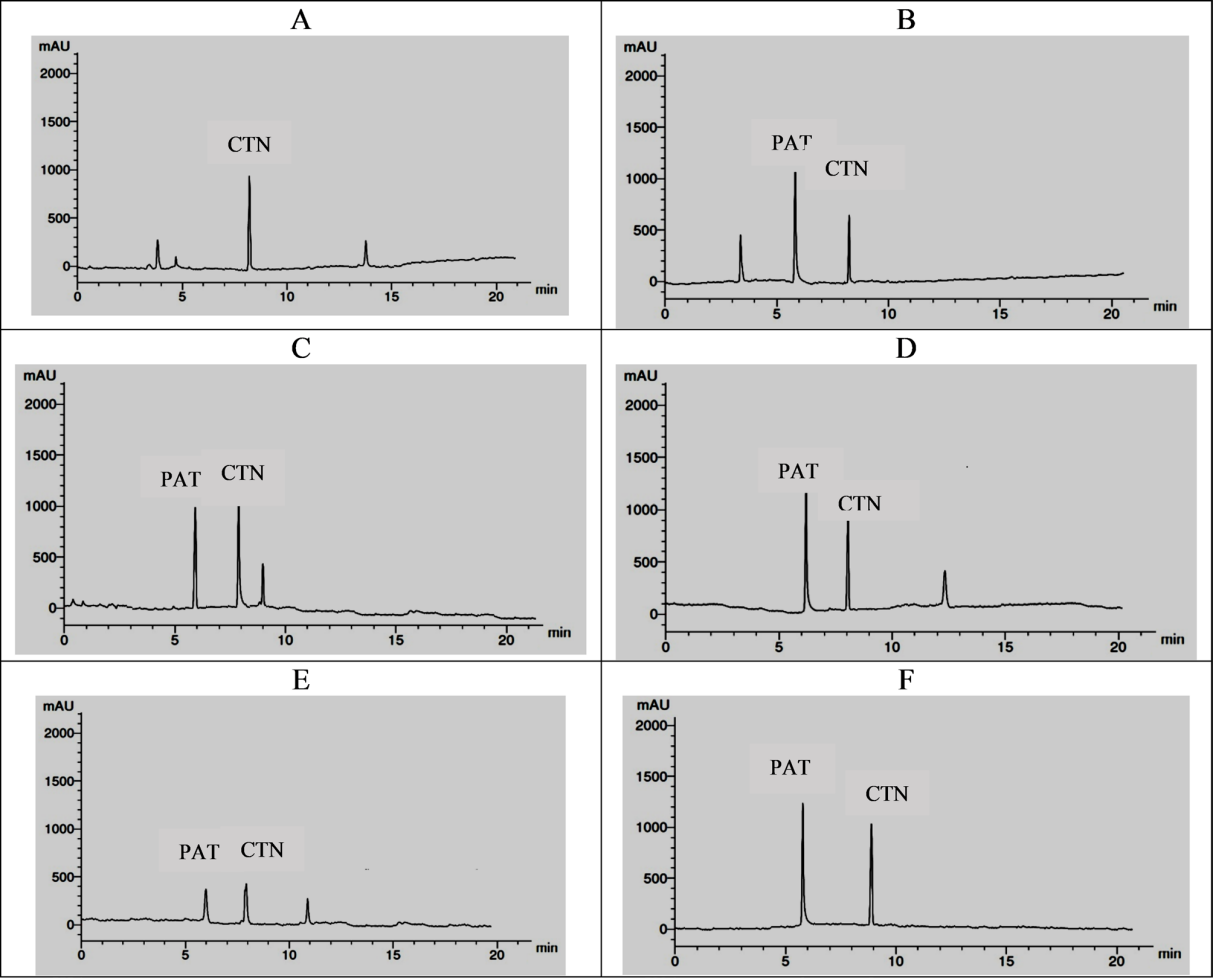
HPLC analysis charts of culture extracts for mycotoxin detection: (**A**) *P. expansum* AP1, (**B**) *P. crustosum* AP2, (**C**) *P. expansum* AP4, (**D**) *P. expansum* AP5, (**E**) *T. atroroseus* AP3, and (**F**) standards

The ability of five isolates (*P. expansum* AP1, *P. crustosum* AP2, *T. atroroseus* AP3, *P. expansum* AP4, *P. expansum* AP5) to produce PAT and CTN mycotoxins was investigated. According to the HPLC analytical findings, these toxins were present in varying amounts in all of the examined fungi's cultural extracts. At varying retention durations, the incidence of these toxins varied from 5210 to 24890 ppb of fungal extracts. Patulin (24180 ppb), which was produced by *P. expansum* AP5, had the greatest amount of toxins, followed by *P. crustosum* AP2 (19360 ppb). The other isolates, however, generated patulin at lower concentrations (5210–18100 ppb). On the other hand, patulin was not detected in the cultural extract of *P. expansum* AP1. The highest value of citrinin (24890 ppb) was detected in the cultural extract of *P. expansum* AP4, followed by *P. expansum* AP5 (19320 ppb). However, the other isolates of fungi secreted citrinin with lower levels (7870-15070 ppb) (Table 3 and Fig. 6).

**Table 3:** HPLC analysis of mycotoxins (ppb)

**Isolates**	**Retention time**	**Patulin**	**Retention time**	**Citrinin**
*P. expansum* AP1	---	ND	8.0	15070
*P. crustosum* AP2	6.0	19360	8.2	8100
*P. expansum* AP4	6.0	18100	8.0	24890
*P. expansum* AP5	6.0	24180	8.0	19320
*T. atroroseus* AP3	6.0	5210	8.0	7870

*ND* Non detectable

## Discussion

Every year, postharvest infections cause significant losses and are a crippling global economic issue Kumar and Kalita [45]. *Penicillium* species are the cause of blue mold, one of the most prevalent postharvest diseases. It affects pome fruit, such as apples, pears, and quinces, both during short-term and long-term storage. Because fruits are succulent, microorganisms can easily infiltrate them Louw and Korsten [47]. When the fungus colonizes damaged fruits, it produces a soft, watery brown lesion with a distinct border, which is the first sign of blue mold degradation. Apart from rot, these fungi also create mycotoxins, which are a significant cause of postharvest loss Bartholomew et al. [11]. Six fungal species from four genera were collected from diseased apple fruits on PDA at 25 °C for the current study. *Talaromyces* and *Penicillium* were the most prevalent fungal genera. Data obtained from early studies were similar to those recorded in our results. They revealed that the genus *Penicillium*, especially *P. expansum*, was the most common fungus isolated from different apple fruits. Additionally, varying frequencies of *Penicillium* species were isolated from a variety of fruits [6, 13, 29, 36, 50, 71]. In this respect, Salau et al. [56] investigated the fruit rot fungi. *Alternaria alternata*, *A. citri*, *Aspergillus niger*, *A. flavus, Aspergillus* sp., *Cladosporium cladosporioides*, *Fusarium solani*, *Fusarium* sp., *Geotrichum candidum, Penicillium* spp., *Phytophthora capsica*, and *Rhizopus stolonifer* were the deterioration pathogens of the postharvest fresh fruits. Saleh and Al-Thani [59] recognized fungi isolated from spoilage fruits and vegetables gathered from the markets in Qatar. Seventy-three fungal isolates were identified. The highest ratio of *Penicillium* was 21.9%, followed by *Rhizopus* (17.8%). Various fruits have variable shelf lives for spoilage. A strong correlation was observed between fungi and the country of origin of fruits. Recently, Hasan and Zanuddin (2020) identified fungi from spoiled fruits sold in markets in Malaysia. Fungi recovered from spoiled banana, mango, and pineapple exhibited the largest frequency in mango (67%), followed by banana (33%). The secluded fungi belonged to *Apergillus*, *Penicillium*, *Fusarium*, and *Cladosporium*. 

Morphological descriptions and measurements for five tested isolates (*P. expansum* AP1, *P. crustosum* AP2, *T. atroroseus* AP3, *P. expansum* AP4, *P. expansum* AP5) were variable. The collected results agree with those documented by multiple workers in various parts of the world, according to a comparison of our data with those found in earlier literature on these species [2, 26, 32, 32, 44] for similar *Penicillium* and *Talaromyces* isolates. These isolates'morphological identity was validated by ITS gene amplification and sequencing. In the GenBank, the five isolates under investigation were tightly clustered with related species. Phylogenetic analysis revealed that the data collected resembled those of other workers [26, 32] and [11]. Olumuyiwa et al. [50] recently verified that there were significant alignments of 93% to 100% in the sequence of the internal transcribed spacer (ITS) region of *Penicillium* species. For *Penicillium* species, the base pair length was 552 with a 99.05% identity rate. The phylogenetic tree was produced using the reference strains from GenBank and the similarity values of the ten strains under study. The isolates with the highest percentage of identity within each morphotype were chosen, and they were compared to the strains with their respective comparable accession numbers.

*P. expansum* AP5 produced the maximum amount of toxins, 24180 ppb, followed by *P. crustosum* AP2 (19360 ppb), according to the current study. However, *P. expansum* AP4 had the highest citrinin value, 24890 ppb, followed by *P. expansum* AP5 (19320 ppb). Fungi producing mycotoxins and causing diseases include genera of *Alternaria*, *Aspergillus*, *Botrytis*, *Cladosporium*, *Colletotrichum*, *Fusarium*, *Geotrichum*, *Penicillium,* and *Rhizopus*. There are no old studies about apple fungi and their mycotoxins in Egypt, however, numerous articles were examined the postharvest fungi of several types of fruits (apple, avocado, banana, dates, guava, lemon, mango, orange, pawpaw, pear, pineapple and strawberry) worldwide [1, 5, 6, 10, 24, 35, 38, 49, 58, 61, 68, 73]. In this respect, Burda [17] investigated 328 samples of apple, pear, and mixed fruit products (such as diced apples, apple pulps, jellies, juices, purees, and sauces) belong 38 Australian producers for their patulin contents using HPLC analysis. Patulin was determined in 75 of 258 concentrate juice and juice samples, with a ratio of 5-50 µg/L. Dombrink-Kmizman and Blackburn [22] evaluated various *Penicillium* species for their ability to secrete patulin in six different culture media. Eleven *Penicillium* strains were tested. The strains belonging to *Penicillium expansum*, *P. griseofulvum* (formerly *P. urticae*), *P. clavigerum*, and *P. coprobium* recovered from apple. Variable limits of patulin production were achieved among different species. *P. griseofulvum* was the highest patulin producer after 96 hrs. *P. expansum* strains were recovered from apple and pear fruits in 106 different cases by Singh and Sumbali (2008). On apples, it was shown that 58.4% of the *P. expansum* strains that were harmful produced both citrinin and patulin, while only 15.6% produced citrinin, 22.1% produced patulin, and 3.9% were nontoxigenic. Ioi et al. [40] reported that patulin is produced by many species of fungi grown on fruits and vegetables. The maximum permissible level of patulin (50 µg/kg) in food was reported by regulatory agencies worldwide [46]. Using Liquid Chromatography Tandem Mass Spectrometry, Hammami et al. [33] examined 45 apple fruits, apple baby meals, and juice samples from different marketplaces in Qatar for fungus and patulin contamination. Twenty-five isolates of *Penicillium* were collected, including one from *P. brevicompactum* and *P. commune* and 23 from *P. expansum*. Every isolate of *Penicillium* that was tested produced between 40 and 100 μg/g of patulin on malt yeast extract agar. In agreement with our results, the ability of 122 *Penicillium expansum* strains isolated from apples in different locations of apple packaging facilities in Lleida, Spain, to produce citrinin was investigated by Viñas et al. [69]. Of the isolates analyzed, 73.2% were isolated from rotten apples, and 46% of them produced citrinin in a lab medium. Dietrich et al. [21] confirmed that there were rare reports about the occurrence of citrinin in foodstuffs. Particularly, the discrepancy between the common detection of the apple rotting *P expansum.* Twenty-four *Penicillium expansum* isolates were obtained from apples grown in British Columbia, Canada, by Abramson et al. [3]. They were cultivated for 28 days at 25 °C in yeast-extract sucrose (YES) to examine the production of citrinin and patulin. Citrinin and patulin were found to be strongly produced by these isolates. The amounts of citrinin, patulin, were 269 and 31 µg/mL, respectively. In total, 91% of the isolates produced citrinin, and 83% produced patulin. Many investigations were performed on patulin and citrinin secretion by fungi recovered from various types of fruits or fruit juices worldwide [7, 18, 19, 33, 46, 48, 53, 70].

## Conclusion

Apple is a perishable fruit. Storage fungi like *Penicillium* (blue mold), able to contaminate fruits and may produce mycotoxins. Fungal analysis for mycotoxin biosynthesis in fruits is important because of transportation and storage conditions sometimes adequate for fungal growth and hazardous metabolites. Patulin and citrinin contamination are harmful to human health and affect the economy of countries worldwide. The environment in warm regions is usually appropriate for fungal growth and contamination. So, it is necessary to investigate the fruits for their fungal and mycotoxins contents. These procedures would contribute to a decrease in food contamination and protect human health. In this study, *P. expansum* AP1, *P. crustosum* AP2, *T. atroroseus* AP3, *P. expansum* AP4, *P. expansum* AP5 were investigated for their potential to produce patulin and citrinin. Selected isolates were able to produce patulin and citrinin with variable degrees. So, in future work, we recommend using a biocontrol technique for controlling fungal infection and mycotoxin production during the transportation and storage of postharvest apple fruits.

## Supplementary Information

Supplementary Material 1.

## Data Availability

The datasets generated and/or analysed during the current study are available in the NCBI GenBank database [https://www.ncbi.nlm.nih.gov/genbank/). ITS rDNA sequences from the five studied strains have been added to the NCBI GenBank database. The accession numbers are as follow: *Penicillium expansum* strain AP1 (PQ859555), *Penicillium crustosum *strain AP2 (PQ859337), *Talaromyces atroroseus* strain AP3 (PQ859549), *Penicillium expansum* strain AP4 (PQ859335), and *Penicillium expansum* strain AP5 (PQ859336).
