# Supplementary material for: Molecular identification and mycotoxins analysis of some fungal isolates from postharvest decayed apple in Qena, Egypt
Source: BMC Plant Biol. 2025 Sep 11;25:1200. doi: 10.1186/s12870-025-07205-2 (PMC12424220; doi:10.1186/s12870-025-07205-2)
Supplement: Supplementary file 1 — Supplementary Material 1. [file 12870_2025_7205_MOESM1_ESM.docx]

**Supplementary Information**

**Gel before cropping (original gel)**

**
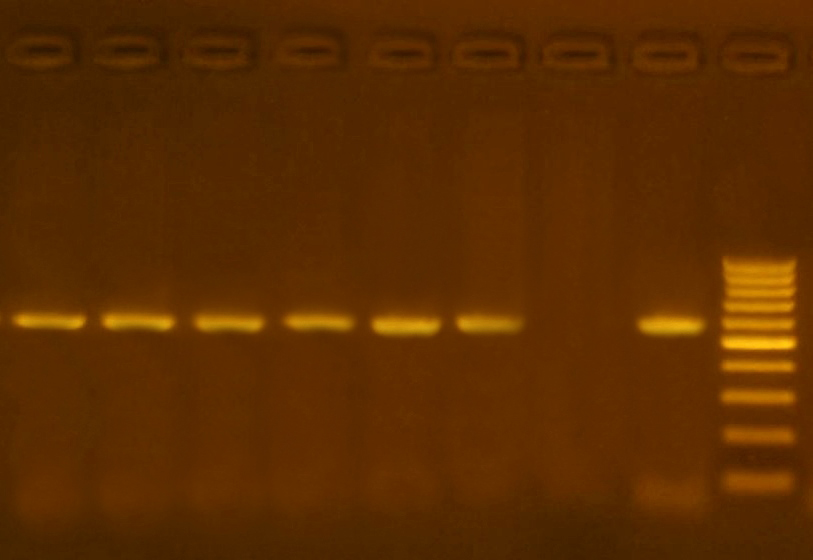
**

Figure 2**.** Agarose gel electrophoresis of PCR amplification of DNA products, PCR amplification of DNA products (~600 bp) using ITS1/ITS4 primer pair. From right to left L—ladder (100–1000 bp); P—positive control consists of a segment of DNA of known size (the same size as the target amplicon, shows that the primers have attached to the DNA strand); N—negative control: a sample without DNA, but contains all essential components of the amplification reaction show if contamination of the PCR experiment with foreign DNA has occurred (from left to right); contaminated isolates (discarded), *P. expansum* AP1, *P. crustosum* AP2,  *T. atroroseus* AP3, *P. expansum* AP4,  *P. expansum* AP5.

**Gel after cropping**

**
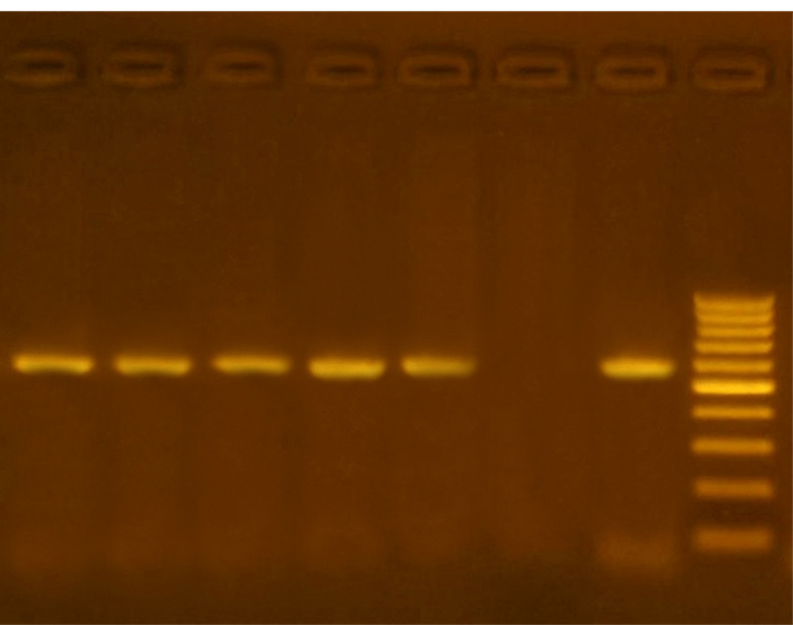
**

Figure 2**.** Agarose gel electrophoresis of PCR amplification of DNA products, PCR amplification of DNA products (~600 bp) using ITS1/ITS4 primer pair. From right to left L—ladder (100–1000 bp); P—positive control consists of a segment of DNA of known size (the same size as the target amplicon, shows that the primers have attached to the DNA strand); N—negative control: a sample without DNA, but contains all essential components of the amplification reaction show if contamination of the PCR experiment with foreign DNA has occurred (from left to right); (4) *P. expansum* AP1, (6) *P. crustosum* AP2, (7) *T. atroroseus* AP3, (9) *P. expansum* AP4, (10) *P. expansum* AP5.

| 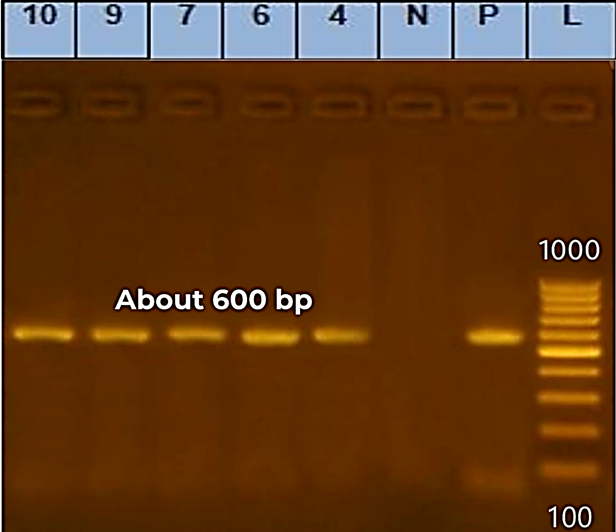  Figure 2**.** Agarose gel electrophoresis of PCR amplification of DNA products, PCR amplification of DNA products (~600 bp) using ITS1/ITS4 primer pair. From right to left L—ladder (100–1000 bp); P—positive control consists of a segment of DNA of known size (the same size as the target amplicon, shows that the primers have attached to the DNA strand); N—negative control: a sample without DNA, but contains all essential components of the amplification reaction show if contamination of the PCR experiment with foreign DNA has occurred (from left to right); (4) *P. expansum* AP1, (6) *P. crustosum* AP2, (7) *T. atroroseus* AP3, (9) *P. expansum* AP4, (10) *P. expansum* AP5. |
| --- |
